# Correction to “The Role of Companion Animals as 'Sentinels' From the One Health Perspective”

**DOI:** 10.1002/vms3.71222

**Published:** 2026-10-12

Kang, S., Y. Chen, and M. Liu. 2026. “The Role of Companion Animals as ‘Sentinels’ From the One Health Perspective.” *Veterinary Medicine and Science* 12, no. 2: e70814. https://doi.org/10.1002/vms3.70814

The authors' affiliations were incorrectly presented as a single combined affiliation. The affiliations in the online version of the article have been corrected accordingly.

Additionally, in the Funding section, the funding information was missing. The updated Funding section is as follows:

Funding

This project was supported by First‐class Discipline Breakthrough Initiative of Hainan University (No. XKTP2025A02)

The online version of this article has been corrected accordingly.

We apologize for this error.

